# Cardiac Recovery: Webcast September 24 2024

**DOI:** 10.14797/mdcvj.1527

**Published:** 2024-12-26

**Authors:** Miguel A. Quiñones, James B. Young, Arvind Bhimaraj, Jane E. Wilcox, Manreet K. Kanwar, Stavros G. Drakos

**Affiliations:** 1 Houston Methodist DeBakey Heart & Vascular Center, Houston, Texas, US; 2 Cleveland Clinic Foundation, Cleveland, Ohio, US; 3 Northwestern University Feinberg School of Medicine, Chicago, Illinois, US; 4 Cardiovascular Institute at Allegheny Health Network, Pittsburgh, Pennsylvania, US; 5 Nora Eccles Harrison Cardiovascular Research and Training Institute, University of Utah Health and School of Medicine, Salt Lake City, Utah, US

**Keywords:** heart failure (HF), myocardial injury, myocardial recovery, HF with improved ejection fraction (HFimpEF), guideline-directed management and therapy (GDMT)

## Abstract

This 61-minute webcast features a conversation about “Cardiac Recovery”—the focus of Issue 20.4. Led by the issue’s editors, the discussion engages the authors on emerging themes and lessons learned while researching and writing the articles. View the video at https://vimeo.com/event/4490511.

## Introduction

This 61-minute webcast (Video 1) features the *Journal*’s editor-in-chief and the issue’s guest editors conversing with three of the issue’s authors on the emerging themes and lessons learned while exploring the article topics related to “Cardiac Recovery.” The following list outlines where to view guests and topics:

**Video 1:** The editors and authors discuss details and explore overarching themes in Issue 20.4, titled “Cardiac Recovery.”

**WELCOME**

Miguel Quiñones, MD, introduces topic (2:09)

Dr. Quiñones introduces guest editors James Young, MD, and Arvind Bhimaraj, MD (4:53)

Dr. Bhimaraj introduces authors and topics (5:56)

**DR. BHIMARAJ MAKES INTRODUCTIONS**

Jane Wilcox, MD (6:33)

Manreet Kanwar, MD (7:03)

Stavros Drakos, MD, PhD (7:30)

Dr. Bhimaraj introduces coeditor Dr. Young (8:15)

Dr. Young opening comments (8:38)

**“CLINICAL PERSPECTIVE OF MYOCARDIAL RECOVERY AND IMPROVEMENT: DEFINITIONS, PREVALENCE, AND RELEVANCE”**

Dr. Bhimaraj begins (11:11)

Dr. Wilcox begins (12:25)

**“LEARNINGS FROM THE 2024 UTAH CARDIAC RECOVERY SYMPOSIUM: A ROADMAP FOR THE FIELD OF MYOCARDIAL RECOVERY”**

Dr. Bhimaraj begins (17:30)

Dr. Drakos begins (18:27)

**“MYOCARDIAL RECOVERY IN CARDIOGENIC SHOCK”**

Dr. Young begins (23:39)

Dr. Kanwar begins (25:00)

Dr. Drakos begins (31:30)

Dr. Young queries (36:16)

Dr. Kanwar responds (36:58)

**CONVERSATION: OUTPATIENT PERSPECTIVE & OPTIONS**

Dr. Quiñones begins (38:22)

Dr. Bhimaraj queries (42:00)

Dr. Wilcox (42:42)

Dr. Kanwar (46:12)

Dr. Drakos (47:22)

**CONVERSATION: DIFFERENCE BETWEEN CARDIAC RECOVERY AND CARDIAC REGENERATION**

Dr. Quiñones begins (51:50)

Dr. Bhimaraj responds (52:24)

Dr. Drakos (54:35)

Dr. Quiñones (57:43)

Dr. Wilcox (59:02)

Dr. Young (59:34)

## Conclusion

Dr. Quiñones wrap-up (1:00:15)

